# Periplocin Induces Apoptosis of Pancreatic Cancer Cells through Autophagy via the AMPK/mTOR Pathway

**DOI:** 10.1155/2022/8055004

**Published:** 2022-07-05

**Authors:** Hewei Zhang, Yongqiang Wang, Yan Zhang, Beishi Zheng, Xiaowu Wang, Jiawei Chen, Bicheng Chen, Gangyin Xie, Lihong Yang

**Affiliations:** ^1^Key Laboratory of Diagnosis and Treatment of Severe Hepato-Pancreatic Diseases of Zhejiang Province, Zhejiang Provincial Top Key Discipline in Surgery, Wenzhou Medical University First Affiliated Hospital, Wenzhou, Zhejiang, China; ^2^Department of Internal Medicine, Woodhull Medical and Mental Health Center, Brooklyn, New York, USA; ^3^Department of Clinical Laboratory, The First Affiliated Hospital of Wenzhou Medical University, Wenzhou, China

## Abstract

Periplocin, a natural compound, has been shown to induce apoptosis in a variety of cancer cells. However, no research has been conducted to demonstrate that Periplocin has a regulatory effect on autophagy. This study is aimed to determine the effect of Periplocin treatment on autophagy in human pancreatic cancer cells, as well as the underlying mechanisms. Pancreatic cancer cells were treated with different concentrations of Periplocin, and real-time cell analysis (RTCA), colony formation assay, and Ki67 immunofluorescence detection were used to determine cell proliferation. Autophagy protein was detected by immunofluorescence and western blotting. Western blotting was also used to detect the caspase family of apoptotic proteins. Flow cytometry and TUNEL staining were used to detect cell apoptosis. Following treatment with Periplocin, the expression of *autophagy* genes was detected using RNA-seq. *In vivo* examination of the effect of Periplocin on autophagy in pancreatic was performed using a xenograft model. Periplocin inhibits the proliferation of CFPAC1 and PANC1 cells and induces autophagy by regulating the AMPK/mTOR pathway. Using the AMPK inhibitor Compound C(CC), both the Periplocin-induced inhibition of cell proliferation and autophagy activation was reduced, which further verified this conclusion. Periplocin inhibits CFPAC1 xenograft tumor growth in nude mice and increases tumor cell autophagy. Collectively, these results have shown that Periplocin promotes autophagy in human pancreatic cancer cells by regulating the AMPK/mTOR pathway.

## 1. Introduction

**Figure 1:**
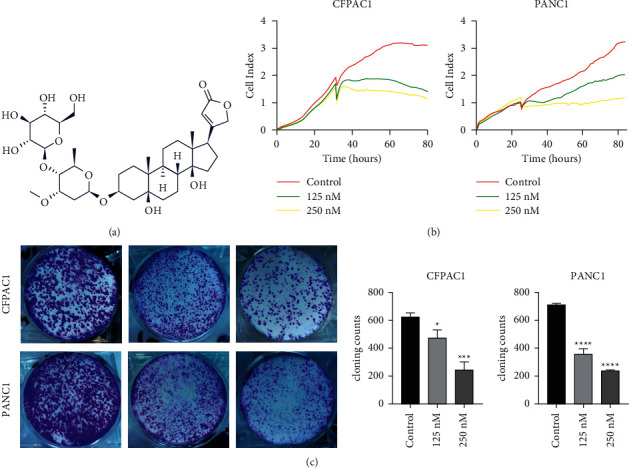
Periplocin inhibits CFPAC1 and PANC-1 cell proliferation. (a) The chemical structure of Periplocin. (b) RTCA following 24 h incubation of CFPAC1 and PANC-1 cells with Periplocin (250 nM, 125 nM) or an equal volume of DMEM media. (c) Colony formation assay following CFPAC1 and PANC-1 cells treated with Periplocin (250 nM, 125 nM) or an equal volume of DMEM medium for 24 h. The data represent the mean ± SE, with ^*∗*^*P* < 0.05, ^*∗∗∗*^*P* < 0.001, ^*∗∗∗∗*^*P* < 0.0001 compared between two groups (*n* = 3).

A natural compound *Cortex periplocae* is the dried root bark of *Periploca sepium*, a member of the Asclepiadaceae family. It is mainly produced in northern China. It has the traditional benefit of strengthening muscles and bones and expelling wind and dampness. It is mostly used to treat waist and knee soreness and weakness, wind-cold dampness, lower limb edema, palpitations, and shortness of breath. Recent research demonstrated that it has numerous benefits, including antitumor, immune-enhancing function, and other effects [1]. Periplocin is a type of monomer that, with further isolation and purification of *C. periplocae*, can inhibit tumor growth (Figure 1(a)). Periplocin can significantly promote the proliferation, migration, and collagen production of fibroblast L929 cells through Na/K-ATPase mediated activation of SRC/ERK and PI3K/Akt signaling pathways, thus promoting wound healing [2]. Periplocin increased the death receptors in gastric cancer cells by activating the ERK1/2-EGR1 pathway, thereby exerting an antitumor effect [3]. Periplocin can also induce the death of esophageal squamous cell carcinoma (ESCC) cells *in vivo* and *in vitro* by downregulating Foxp3, increasing the expression of DR4/DR5, and increasing the cells' sensitivity to trial [4]. Lohberger et al. found that Periplocin specifically kills liposarcoma cells by activating apoptotic death receptor-mediated pathways [5]. Our previous study demonstrated that Periplocin inhibited pancreatic cancer cell proliferation and induced apoptosis by activating the AMPK/mTOR pathway and inhibiting p70S6 kinase. Periplocin inhibited the growth of CFPAC1 in nude mouse xenografts *in vivo* [6]. There are mainly three signaling pathways that regulate autophagy in pancreatic cancer: (1) AMPK-mTORC1 pathway [7], (2) IPI3K/Akt/mTORC1 pathway [8], and (3) RAS/RAF/MEK/ERK pathway [9]. However, the effect of Periplocin on autophagy has not been investigated in pancreatic cancer.

Pancreatic cancer has a poor prognosis because of its vague early symptoms and late detection. It is now one of the cancers with the highest mortality rate worldwide [10]. Clinical treatment strategies for pancreatic ductal adenocarcinoma (PDAC) are presently under discussion. Conventional treatment approaches such as surgical resection, chemotherapy, and radiotherapy are the only options available; nevertheless, the therapeutic outcome is unsatisfying, with a 5-year survival rate of less than 7% [11]. Patients diagnosed with PDAC have a 10%–15% chance of being cured through surgical excision, which is the only treatment option now available. Clinical trials of adjuvant chemotherapy administered after surgery include the multicenter phase III randomized controlled clinical study Jaspac01 carried out in JAPAN [12]. The European ESPAC-4 study [13] compared the efficacy of gemcitabine combined with capecitabine and gemcitabine monotherapy. This comparison suggests that further research is needed to determine whether chemotherapy regimens with better postoperative adjuvant chemotherapy efficacy, such as *S* − 1 or gemcitabine combined with capecitabine, can still improve the survival of patients. Simultaneously, chemotherapeutic agents are a common source of drug resistance. Consequently, there is an urgent need for the development of new PDAC treatment regimens and effective drugs.

Studies have shown that when cells are exposed to hypoxia, nutrient starvation, endoplasmic reticulum stress, ultraviolet radiation, and other stressful conditions, autophagy can be induced [14]. Other studies have demonstrated that AMPK induces autophagy in response to numerous cellular stress stimuli [15]. Pancreatic ductal adenocarcinoma is the most common pathological type of pancreatic cancer, and K-ras mutations can be detected in more than 90% of PDAC. The activation of RAS can significantly improve the level of autophagy. It promotes the proliferation, migration, and cell metabolism of pancreatic cancer cells [16, 17]. Autophagy plays a role in both tumor inhibition and tumor cell survival. Therefore, the role of autophagy in the growth of pancreatic cancer cells mediated by Periplocin through the AMPK/mTOR pathway requires to be investigated. Therefore, in this study, we investigated the effect of Periplocin on autophagy in pancreatic cancer cells and further explored the role of autophagy in the Periplocin's activation of the AMPK/mTOR pathway to inhibit the growth of pancreatic cancer cells.

## 2. Materials and Methods

### 2.1. Reagents and Antibodies

Periplocin (CAS No.: 13137-64-9, Formula: C_36_H_56_O_13_) and Compound C (CAS No.: 866405-64-3, Formula: C_24_H_25_N_5_O) were obtained from Selleck (USA) and prepared as 10 mM stock solutions in DMSO. The primary antibodies were purchased from Cell Signal Technology (Boston, USA) and included the following: anti‐GAPDH (#5174), anti‐P62 (#39749), anti‐LC3B (#2775), anti‐AMPK (#2532), anti‐p‐AMPK (Thr172, #2535), anti‐mTOR (#2972), anti‐p‐mTOR (Ser2448, #2971), anti‐cleaved‐caspase3 (#9667) and anti‐Ki67 (#12075). Secondary antibodies included antimouse (Zen bio, Chengdu, China), antirabbit antibodies (Zen bio, Chengdu, China), and CoraLite488 conjugated fluorescent secondary antibody (Cat No.SA00013-2, Proteintech, China).

### 2.2. Cell Culture

Human cancer cell lines including CFPAC1 and PANC1 were purchased from the American Type Culture Collection (ATCC, USA). All cell lines were maintained in Dulbecco's Modified Eagle Medium (DMEM, GIBCO, USA) supplemented with 10% fetal bovine serum (FBS, GIBCO, USA), 100 U/mL penicillin, 100 *μ*g/mL streptomycin (GIBCO, USA) at 37°C in a humidified incubator with 5% CO_2_.

### 2.3. Real-Time Cell Analyses

The RTCA analysis was performed using an xCELLigence MP system (ACEA Biosciences, San Diego, CA, USA). For background measurements, 50 *μ*L of cell culture media was added to the wells. CFPAC1 and PANC1 cells were seeded into E-plate 16 (00300600890, ACEA, USA). The cells (∼5000/well) were then plated in fresh media to a final volume of 150 *μ*l. Periplocin (0, 125, and 250 nM) was supplemented to the medium 20 hours after cell seeding. The RTCA-DP software (Roche Diagnostics GmbH) was used to record all data.

### 2.4. Clone Formation Assay

Clone formation assay was performed as previously described [18]. Briefly, 1000 cells were inoculated into 6-well plates. The cells were then treated with Periplocin (0, 125, and 250 nM) for 24 h. The clones were visible to the naked eye after 14 days. The samples were fixed with 4% paraformaldehyde for 30 min and stained with 0.5% crystal violet for 20 min. Photographs were taken using a digital camera, and the number of colonies was counted and recorded. The experiments were carried out in triplicates.

### 2.5. Immunofluorescence Staining Analysis

The immunofluorescence analysis was performed as previously described [19]. Anti-Ki67 and anti-LC3B were used as primary antibodies. CoraLite® 488 goat antirabbit was used as a secondary antibody. The nuclei were stained using 4′, 6-diamidino-2-phenylindole (DAPI, Sigma-Aldrich, Missouri, US). The images were captured using an immunofluorescence microscope. The quantitative colocalization analysis was performed using Image-Pro Plus (version 6.0, Media Cybernetics).

### 2.6. Flow Cytometric Analysis of Apoptosis

Flow cytometry with the FITC-Annexin V Apoptosis Detection Kit (556547, BD Pharmingen) was used to measure apoptosis. After 24 h treatment with Periplocin (0, 125, and 250 nM), cells were washed with cold phosphate‐buffered saline and collected. The cells were then resuspended in 100 *μ*L binding buffer and 5 *μ*L of Annexin V-FITC and propidium iodide (PI) was added. Following a 15 min incubation at room temperature (25°C) in the dark, 400 *μ*L of binding buffer was added to each sample. The FACSCalibur system (BD Biosciences, San Jose, CA, USA) was used to assess the apoptosis rates. Data analysis was performed using FlowJo software (FlowJo, version 10.0.7).

### 2.7. Western Blotting Analysis

To isolate proteins from pancreatic cancer cells, RIPA buffer (Beyotime, Shanghai, CN) supplemented with protease and phosphatase inhibitors (Beyotime, Shanghai, CN) was used to lyse the cells. The lysed tissues and cells were further ultrasonically broken and centrifuged at 12000 rpm for 15 min at 4°C to separate the supernatant from the cell debris. Proteins in the supernatant were quantified using a bicinchoninic acid (BCA) kit (Beyotime, Shanghai, CN). The proteins were subsequently separated in 10% sodium dodecyl sulfate-polyacrylamide gels, and the resultant bands were transferred onto PVDF membranes. The membranes were first blocked with 5% skim milk on a shaker for 1 hour and then incubated overnight at 4°C with primary antibodies (1 : 1000, anti-GAPDH, anti-AMPK*α*, anti-p-AMPK, anti‐mTOR, anti‐p‐mTOR, anti‐cleaved‐caspase3, anti‐P62, and anti‐LC3B). The membranes were then washed with TBST to remove excess primary antibodies before being incubated at room temperature for 1 h with the corresponding secondary antibody (1 : 5000). Subsequently, the protein bands were visualized using the chemiluminescence method, and densitometry analysis was performed using Image-Pro Plus software.

### 2.8. Immunohistochemistry Staining

During the final week, the nude mice were sacrificed and xenograft tumors were isolated. Then tumors were fixed with 10% formaldehyde for 24 h. Tumors tissues were paraffin‐embedded and cut into 4-*μ*m-thick sections. The tissue sections were deparaffinized and rehydrated, sealed with 3% hydrogen peroxide, and repaired with 5% goat serum antigen. The sections were then incubated at 4°C overnight with Ki67(1 : 200) and LC3B primary antibodies (1 : 200). They were then incubated with a goat antirabbit secondary antibody for 60 min at 37°C and observed with diaminobenzidine (DAB). The cell nuclei were then stained with hematoxylin for 3 min at 25°C. Representative tissue images were captured using a light microscope and the positive regions were analyzed using the Image-Pro Plus 6.0 software.

### 2.9. Evaluation of Tumor Cell Proliferation, Autophagy, and Apoptosis *In Vivo*

Male BALB/c nude mice (6 weeks old, 19–21 g) were obtained from the Experimental Animal Center of Wenzhou Medical University. All nude mice were housed with 12 h-light and 12 h-dark cycles at 24 ± 2°C. CFPAC1 cells (5 × 10^6^ cells) were suspended in a 200 *μ*L medium and injected into the back of each mouse. When the tumor size reached approximately 100 mm^3^, nude mice were randomly divided into two groups (*n* = 5/group) and administered with daily intraperitoneal injections of either 0.1%DMSO (control group) or Periplocin 25 mg/kg. With the same frequency, the tumor volumes were observed. The volume was calculated as follows: tumor volume = length × width^2^ × 0.5. After 11 days, the tumors were collected, weighed, and photographed. Hematoxylin and eosin (H&E) staining was performed for histopathological analysis. Western blotting was performed to detect the autophagy protein LC3BII/LC3BI and apoptotic protein cleaved-caspase3. TUNEL detection kit (Roche, Indianapolis, IN) was performed according to the manufacturer's instruction and tumor apoptosis was analyzed using a fluorescence microscope (Nikon, Japan). All animal experiments and experimental procedures were performed following the guidelines of the Ethics Committee of Wenzhou Medical University.

### 2.10. RNA-Seq

The data set consisted of six PANC-1 cell samples that were treated with 0 and 125 nM Periplocin for 24 h in three biological replicates. Total RNA was isolated and purified using TRIzol (Invitrogen). RNA samples were then sent to BGI (Shenzhen, China). The resulting RNA-seq library was sequenced on a BGISEQ-500 sequencer. The differentially expressed mRNAs were selected with fold change >2 or <0.5 and *P*-value < 0.05.

### 2.11. Statistical Analysis

Statistical analysis was performed using GraphPad Prism software (version 8.0, GraphPad Software, CA, USA). All data were presented as mean ± SD of three independent experiments. The student's *t*-test and one-way analysis of variance (ANOVA) were used to determine statistical significance. *P* ≤ 0.05 was considered statistically significant.

## 3. Results

### 3.1. Periplocin Inhibits Proliferation and Increases Autophagy in PDAC Cell Lines

**Figure 2:**
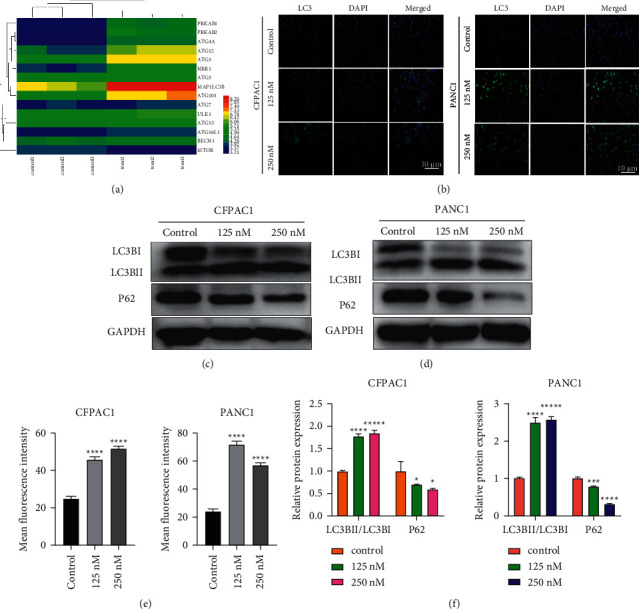
Periplocin induces autophagy in both CFPAC1 and PANC-1 cells. (a) Cluster heat map of autophagy genes. Downregulated genes are shown in blue, and upregulated genes are shown in red. (b) LC3B Immunofluorescence when CFPAC1 and PANC-1 cells were treated with Periplocin (125 nM, 250 nM) or an equal volume of DMEM media for 24 h. (c, d) The effects of Periplocin on the expression of p62 and LC3BII/I proteins in CFPAC1 and PANC-1 cells, as determined by Western blotting analysis. (e) Statistical analysis of the mean fluorescence intensity of the LC3 protein. (f) Western blot quantitative statistical analysis of the LC3B and P62 proteins. The data represent the mean ± SE, with ^*∗*^*P* < 0.05, ^*∗∗∗*^*P* < 0.001, ^*∗∗∗∗*^*P* < 0.0001 compared between two groups (*n* = 3).

RTCA and colony formation assay was performed to determine the effect of Periplocin on the proliferation of pancreatic cancer cells. As shown in Figure 1(b), Periplocin (125 nM, 250 nM) treatment significantly inhibited the growth of pancreatic cancer cells. Periplocin also significantly decreased colony formation in CFPAC1 and PANC-1 cells (Figure 1(c)). According to these findings, Periplocin inhibited the proliferation of pancreatic cancer cells in a concentration and time-dependent manner. To further investigate the effect of Periplocin on autophagy in pancreatic cancer cells, we analyzed the RNA-seq results of PANC1 cells treated with 125 nM Periplocin.  Figure 2(a) shows the cluster heat map of autophagy-related genes. The heat map showed that *PRKAB*, *LC3B*, and *ATG* genes were significantly upregulated, while in contrast, the *mTOR* gene was significantly downregulated. To verify that Periplocin promotes autophagy in pancreatic cancer cells, CFPAC1 cells and PANC1 cells were treated with different concentrations of Periplocin (0.125 nM, 250 nM) for 24 hours, and the expression of autophagy regulatory proteins was detected by Immunofluorescence and Western blotting. Immunofluorescence results (Figures 2(b) and 2(e)) revealed that in the Periplocin-treated group, there were significantly more LC3B green fluorescent spots scattered throughout the cytoplasm. In the control group, green LC3B fluorescence was randomly distributed throughout the cytoplasm. According to Figures 2(c), 2(d), and 2(f), the expression of LC3B protein in CFPAC1 and PANC1 cells increased significantly and was positively correlated with drug concentration. In contrast, the expression of P62 protein was significantly reduced, and negatively correlated with drug concentration. In conclusion, Periplocin promotes autophagy in pancreatic cancer cells.

### 3.2. Periplocin Induces Apoptosis of PDAC Cell Lines

**Figure 3:**
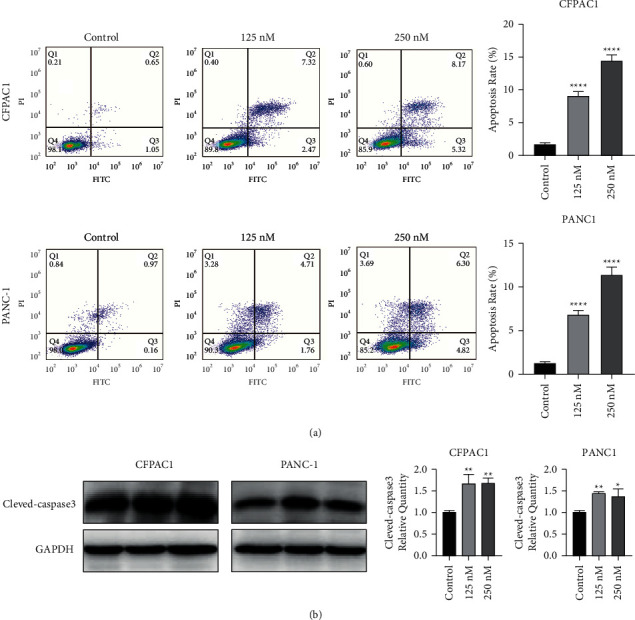
Periplocin induces apoptosis in both CFPAC1 and PANC-1 cells. (a) Flow cytometry analysis of apoptosis in CFPAC1 and PANC-1 cells treated with Periplocin (250 nM, 125 nM) or an equal volume of DMEM media for 24 h. (b) Expression of cleaved-caspase3 in CFPAC1 and PANC-1 cells following treatment with 250 nM, 125 nM periplocin, respectively. The data represent the mean ± SE, with ^*∗*^*P* < 0.05, ^*∗∗∗*^*P* < 0.001, ^*∗∗∗∗*^*P* < 0.0001 compared between two groups (*n* = 3).

To explore the effect of Periplocin on the apoptosis of pancreatic cancer cells, we assessed the effects of Periplocin on pancreatic cancer cell apoptosis. As shown in Figure 3(a), Periplocin treatment significantly increased the proportion of apoptotic cells compared to the control group. Western blot analyses further confirmed that apoptosis-related protein cleaved-caspase-3 expression was upregulated after Periplocin treatment (Figure 3(b)).

### 3.3. Periplocin Regulates the AMPK/mTOR Signaling Pathways in Pancreatic Cancer Cells

**Figure 4:**
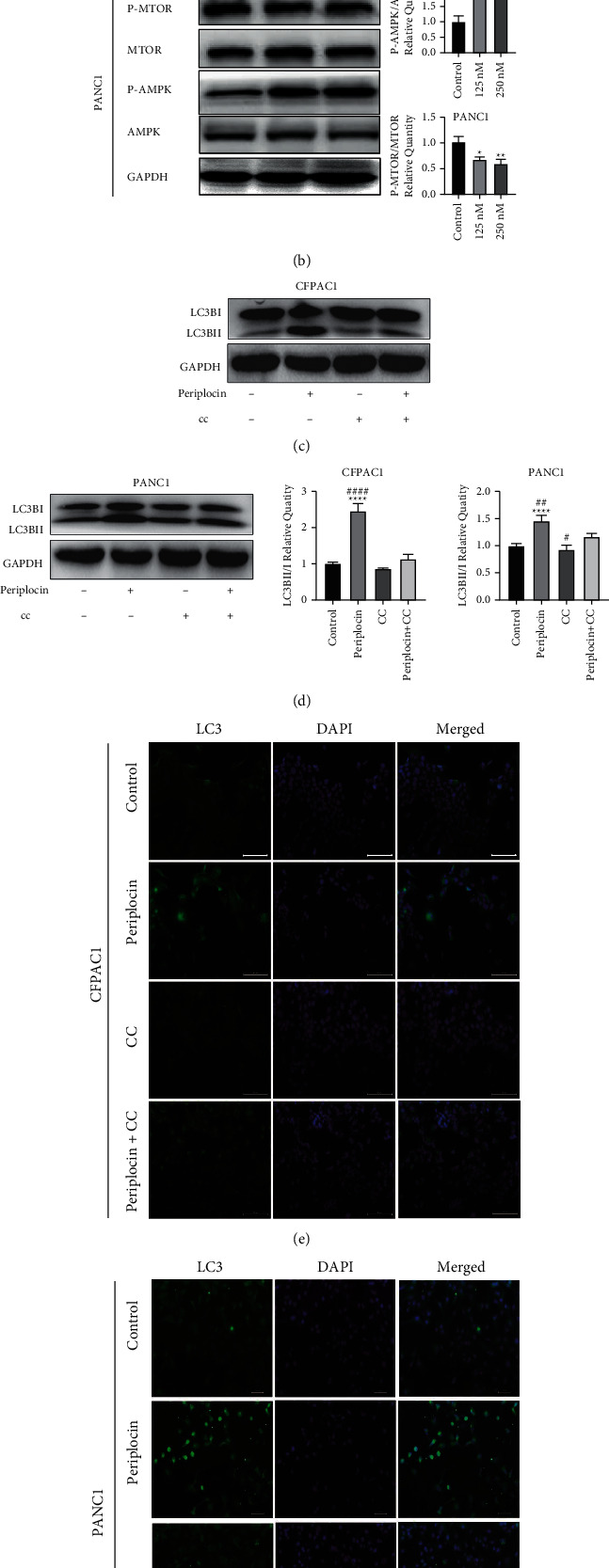
Periplocin-induced autophagy is dependent on AMPK/MTOR signaling. (a, b) After 24 h of periplocin treatment (125 nM, 250 nM). The expression of AMPK/MTOR signaling pathway proteins detected by Western blotting analysis. (c, d) Expression of LC3BII/I of CFPAC1 and PANC-1 cells after treatment with 125 nM periplocin and/or 5 *μ*M CC detected by Western blotting analysis. (e) Expression of LC3B of CFPAC1 and PANC-1 cells after treatment with 125 nM periplocin and/or 5 *μ*M CC detected by immunofluorescence. The data represent the mean ± SE, with ^*∗*^*P* < 0.05, ^*∗∗∗*^*P* < 0.001, ^*∗∗∗∗*^*P* < 0.0001 compared between two groups and ^#^*P* < 0.05, ^##^*P* < 0.01 and ^####^*P* < 0.0001 compared with the group treated with Periplocin and CC (*n* = 3).

AMPK and mTOR are the primary autophagy regulators. AMPK positively regulates autophagy by inhibiting the activity of mTOR complex 1 (mTORC1) directly and indirectly. Western blotting was utilized to detect the protein expression of the AMPK-mTOR pathway to further verify the heat map results of the signal pathway. In Periplocin-treated CFPAC1 and PANC-1 cells (Figures 4(a) and 4)(b), increased p-AMPK expression and decreased p-mTOR expression were observed. These results indicated that Periplocin may promote increased autophagy in pancreatic cancer cells by regulating the AMPK-mTOR signal axis, thereby inhibiting pancreatic cancer cell proliferation and inducing apoptosis.

### 3.4. AMPK/mTOR Signaling Pathway Inhibition Reduces Periplocin-Induced Autophagy and Proliferation Inhibition

**Figure 5:**
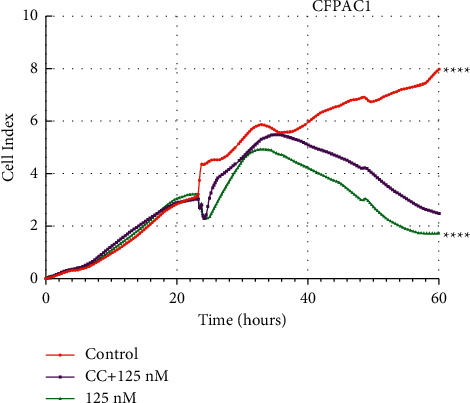
CC reverses the inhibitory effect of Periplocin on CFPAC1 cells. The proliferation of CFPAC1 cells treated for 24 h with 125 nM Periplocin and/or 5 *μ*M CC was assessed by RTCA. The data represent the mean ± SE, with ^*∗∗∗∗*^*P* < 0.0001 compared with the control.

To determine whether Periplocin induces autophagy and proliferation inhibition in pancreatic cancer by increasing AMPK phosphorylation, PDAC cells were treated with a commonly used AMPK inhibitor, compound C (5 *μ*M), and Periplocin (125 nM). As shown in Figures 4(c)–4(f), the expression level of LC3BII/LC3BI was significantly lower in the Compound C group than in the Periplocin group. RTCA results (Figure 5) demonstrated that Compound C partially restored the suppressive effect of Periplocin on proliferation (Figure 5). The above results indicated that Compound C reversed Periplocin induced an increase in autophagy and inhibition of pancreatic cancer cell proliferation through AMPK suppression.

### 3.5. Periplocin Inhibits Pancreatic Cancer Xenograft Tumors Development and Mediates Autophagy *In Vivo*

**Figure 6:**
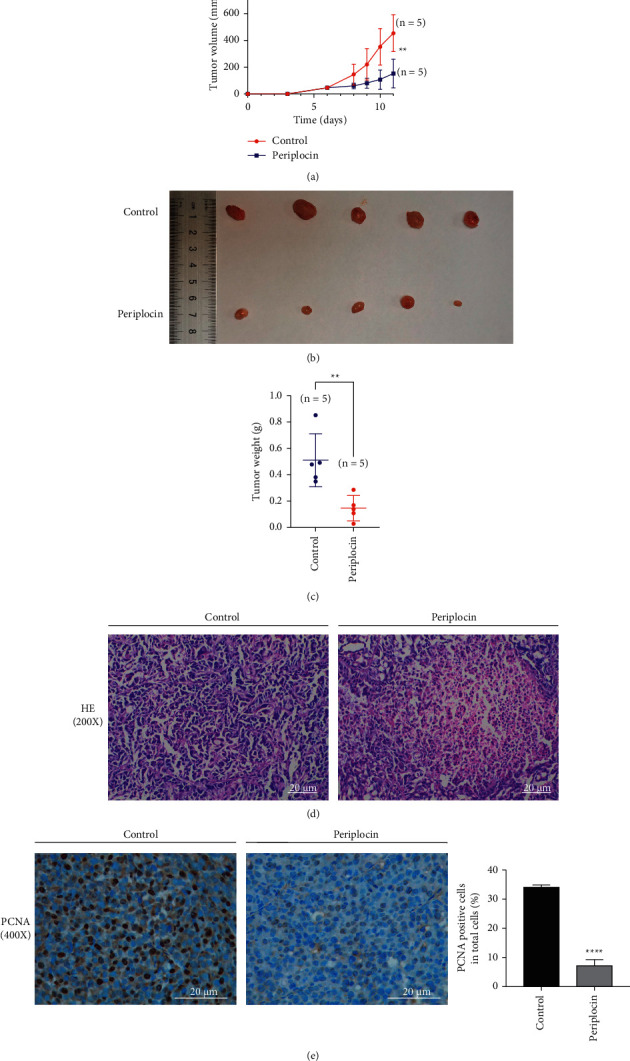
Periplocin inhibited tumor growth in nude mice. (a) Growth curve of *in vivo* tumor growth (*n* = 5; ^*∗∗*^*P* < 0.01). (b) The effect of Periplocin on the tumorigenesis of nude mice was measured in a xenograft trial. (c) Comparison of tumor tissue weight between the control and Periplocin groups (*n* = 5; ^*∗∗*^*P* < 0.01). (d) HE staining of tumors, scale bar = 20 *μ*m (200x). (e) Immunohistochemistry staining of PCNA in tumors *in vivo*, scale bar = 20 *μ*m (400x).

**Figure 7:**
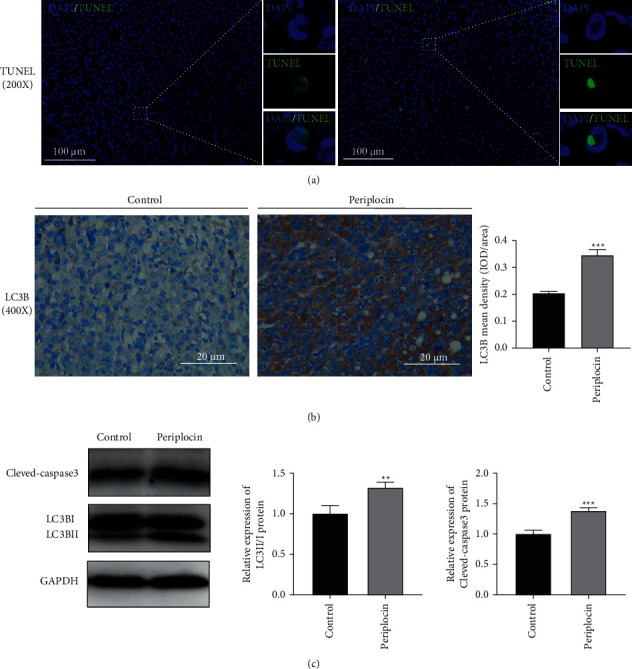
Periplocin increases autophagy and apoptosis in pancreatic cancer tumors *in vivo*. (a) TUNEL staining of CFPAC1 tumor sections following various treatments, scale bar = 100 *μ*m. (b) Immunohistochemistry staining for LC3B in tumors *in vivo*, scale bar = 20 *μ*m (400x). (c) Expression of cleaved-caspase3 and LC3BII/I in the CFPAC1 tumor model as detected by Western blotting following different treatments.

To explore the anticancer effect and mechanism of Periplocin *in vivo*, we established a CFPAC1 cell subcutaneous tumor model in nude BALB/c mice. The tumor growth curve demonstrated that the volume of subcutaneous tumors in the Periplocin injection group was significantly less than in the control group (Figure 6(a)). Consistent with the observations on tumor volume, the tumor weight also significantly decreased (Figures 6(b) and 6(c)).  Figure 6(d) shows the H&E staining of the tumor tissue. Comparing the Periplocin group to the control group, tumor tissue in the Periplocin group exhibited necrotic regions. Immunohistochemistry results (Figure 6(e)) revealed a significant decrease in the proportion of proliferative protein PCNA-positive cells in the subcutaneous tumor tissue of nude mice treated with Periplocin compared to the control group. TUNEL staining was used to assess apoptosis. The apoptotic rate of subcutaneous tumors in nude mice treated with Periplocin was significantly increased than in the control group (Figure 7(a)). Immunohistochemical results (Figure 7(b)) revealed that the expression of LC3B protein in Periplocin-treated tumor tissues was significantly higher than that of the control group. As shown in Figure 7(c), Western blotting revealed that the expression of the apoptotic proteins cleaved-caspase3 and LC3BII/LC3BI in the subcutaneous tumor tissue of nude mice in the Periplocin group was significantly higher than in the control group. In summary, these results indicated that Periplocin induced autophagy, and apoptosis, and inhibited pancreatic cancer cell growth *in vivo*.

## 4. Discussion

In this study, RNA-seq sequencing analysis of PANC-1 cell samples treated with 125 nM Periplocin and untreated PANC-1 cell samples for 24 h revealed that differentially expressed genes were primarily concentrated in the AMPK/mTOR pathway and the autophagy process. In the treatment group, autophagy-related genes were significantly upregulated, as indicated by the clustering heat map. Western blot analysis confirmed that the autophagy marker protein LC3B increased in a concentration-dependent manner, while the expression of P62 decreased in a concentration-dependent manner. In addition, LC3B antibody immunofluorescence staining was utilized to detect the formation of fluorescent spots, and the formation of autophagosomes was increased. The combination of Periplocin and the AMPK inhibitor CC can reduce the appearance of LC3B antibody fluorescent spots. Western blot analysis revealed that the expression level of LC3B-II/I in the group treated with AMPK inhibitor CC was significantly lower than in the group treated with Periplocin alone. Following the administration of the final dose of Periplocin associated with CC, the PANC1 cell growth index partially recovered, and animal studies confirmed this. These findings imply that Periplocin may inhibit cell growth by inducing autophagy transition activation in pancreatic cancer cells via the AMPK/mTOR pathway.

Discovered in 1963, autophagy is a lysosomal-mediated degradation of nonessential or damaged cell components [20]. It refers to the formation of autophagosomes by including some cytoplasmic and intracellular organelles, proteins, and other elements into the cytoplasmic bilayer membrane, as well as the fusion of an autophagosome with a lysosome to form autophagy-lysosome and the degradation of its contents [21–23]. This study revealed that Periplocin can induce autophagy transition activation in pancreatic cancer cells, thereby inhibiting cell growth. Previous research has demonstrated that excessive autophagy in several pancreatic cancer cells can lead to cell growth inhibition. Jia et al. [24] found that the levels of p8 and its targets p53 and PKC-*α* were increased in fisetin-treated PANC-1 cells and that p8 inhibited the fisetin-induced autophagy. Thus, Panc1 cell proliferation was inhibited. Zhang et al. [25] discovered that silibinin can also promote autophagy and inhibit pancreatic cancer cell proliferation. JNK/SAPK inhibitors can reverse these biological effects of silibinin.

Xu et al. [26] discovered LZ1, a peptide derived from the venom of antimicrobial peptides. *In vitro* and *in vivo*, LZ1 significantly inhibited pancreatic cancer cell proliferation by inducing autophagic cell death. LZ1 exhibits an action through binding to nuclear protein, and nuclear protein frequently increased cell surface expression of pancreatic cancer. LZ1 binding triggers the degradation of surface-expressed nucleoproteins. This activates AMPK, which then suppresses mTORC1 activity and induces autophagy flux. However, the results of this study are inconsistent with the following literature: in the subcutaneous xenotransplantation model of pancreatic cancer in nude mice, resveratrol inhibits the expression of −1 (NAF-1), which inhibits autophagy [27, 28]. Ancistrolikokine E3, an effective early inhibitor of the autophagy pathway in PANC-1 pancreatic cancer cells, significantly inhibits the migration and colony formation of PANC-1 cells in a concentration-dependent manner [29]. Matrine inhibits KRAS mutant pancreatic cancer growth by inhibiting autophagy-mediated energy metabolism and mitochondrial energy production [30]. Alantolactone TFEB injury by targeting the autophagolysosome pathway inhibits autophagy-induced apoptosis of pancreatic cancer cells and pancreatic cancer cells enhanced chemosensitivity caused by oxaliplatin [31]. Autophagy limits the inhibition of pancreatic tumor growth and tumorigenesis [32]. Studies have demonstrated that autophagy may antagonize the apoptotic effects by promoting cell survival. Autophagy has also been demonstrated to play an essential role in tumor cell survival. In cancer cells, autophagy is used to respond to cellular stress [33–35]. These stressors activate autophagy to recover ATP and maintain the viability of cancer cells. However, the present study reveals that Periplocin induces autophagy. However, whether it stimulates apoptosis requires to be further elucidated. In addition, the precise mechanism should also be further investigated, which will provide novel strategies for preventing and treating pancreatic cancer.

Several autophagy-inducible drugs, including Tamoxifen and mTOR protein inhibitors, have been used in the clinical treatment of various malignant tumors. Other drugs undergoing preclinical or clinical testing include those that inhibit tumors via protein inhibitors such as caspase. The drug development of autophagy signal-regulating pathways holds promising potential in the treatment of pancreatic cancer.

## 5. Conclusion

In conclusion, our research confirms that Periplocin is an effective compound against pancreatic cancer cells. Periplocin increased autophagy in pancreatic cancer cells by activating the AMPK/mTOR signaling pathway, as determined by measuring the protein expression levels of significant signaling pathways.

## Data Availability

Please contact the first/corresponding author directly for the availability of data.

## Abbreviations

RTCA: Real-time cell analysis
CC: Compound C
ESCC: Esophageal squamous cell carcinoma
PDAC: Pancreatic ductal adenocarcinoma
ATCC: American type culture collection
DMEM: Dulbecco's modified eagle medium
FBS: Fetal bovine serum
ANOVA: Analysis of variance.
